# No one left to cope alone (NOLA): protocol for the development of a postdiagnostic intervention to support people newly diagnosed with dementia

**DOI:** 10.1136/bmjopen-2024-091241

**Published:** 2024-10-11

**Authors:** Charlotte R Stoner, Helen Durgante, Linda Birt, Aimee Spector

**Affiliations:** 1 Centre for Chronic Illness and Ageing, Institute for Lifecourse Development, School of Human Sciences, University of Greenwich, London, UK; 2 Federal University of Pelotas, Pelotas, Brazil; 3 University of Leicester, Leicester, UK; 4 Department of Clinical, Educational, and Health Psychology, University College London, London, UK

**Keywords:** Dementia, Dementia, QUALITATIVE RESEARCH, Quality of Life

## Abstract

**Abstract:**

**Introduction:**

Receiving a diagnosis of dementia is a seminal moment in many people’s lives. It can be associated with anger and grief for both the person with dementia and their network. Despite this, there is no provision for emotional support to help people affected by dementia manage the impact of receiving that diagnosis. As such, a postdiagnostic intervention to help people process and adjust to a diagnosis of dementia is needed. This protocol describes the initial work to be undertaken as part of a Programme Development Grant. The aims are to synthesise evidence for existing interventions, understand the implementation context and establish an advisory board.

**Methods and analyses:**

Phase 1 will consist of two systematic reviews to synthesise research evidence for existing interventions in related areas. Phase 2 will consist of two qualitative research studies. Study 1 is a UK-wide qualitative survey to understand the current lived experience of receiving a diagnosis and postdiagnostic support. Study 2 is a local qualitative study in which three groups of stakeholders will be asked about the perceived barriers and facilitators to implementing a postdiagnostic intervention in Natuional Health Services (NHS). In Phase 3, an advisory group of people living with dementia, carers and other professionals will be established to provide advice and feedback and contribute to the codevelopment of the initial intervention.

**Ethics and dissemination:**

Health Research Authority, 15 July 2024. All data will be held in accordance with North East London NHS regulations, who act as sponsor of this development work. We will engage with policy professionals in Study 2 (Phase 2) and through this network disseminate our findings to facilitate policy change. The use of coproduction to ensure people with dementias voices are heard throughout this work will result in impact in health and well-being.

**STRENGTHS AND LIMITATIONS OF THIS STUDY**

- Extensive public and patient engagement, making use of an Advisory Board including people living with dementia and caregivers, will ensure that all work is informed by the lived experience of dementia and result in an evidence-based, acceptable intervention for those affected.
- Utilisation of the consolidated framework for implementation research and research with key policy professionals and decision makers will result in a thorough investigation of the implementation context during development work.
- Initial work is developmental and, while will result in key components being identified, it will not result in a fully developed intervention ready for evaluation.
- Further development and a randomised controlled trial will be contingent on a successful further grant application.

## Introduction

 Approximately 434 307 people in England have been diagnosed with dementia. During COVID-19 diagnosis levels dropped by at least 6.3% from March 2020 to February 2021, with 33 500 diagnoses needed to get back to prepandemic levels.^1^ As these individuals present to services in the coming years, there is likely to be an unprecedented demand, especially as people with undiagnosed dementia often only present to services during a crisis.^2^

The National Dementia Strategy^2^ emphasised the need for early and improved rates of diagnosis. The benefits of this for improving access to care are paramount; however, the initial impact of this diagnosis on the individual could be poorly considered. Receiving a diagnosis of dementia has been described as ‘traumatic’^3^ and can have a significant, detrimental effect on identity and relationships.^4^ People with dementia have suggested that, as a result of the diagnosis, they no longer see themselves as ‘normal’,^5^ and this has been associated with feelings of anger, grief and loss,^6^ with carers often experiencing anticipatory grief reactions.^7 8^

Despite widespread evidence suggesting a diagnosis of dementia is a significant life event and national guidelines confirming the emotional impact of a diagnosis of dementia,^9^ the provision of postdiagnostic support for emotional well-being in National Health Service (NHS) memory clinics can be absent or unequally distributed.^10^ Further, these unmet care needs are likely to be increasing disease burden for people with dementia who are ‘Left to Cope Alone’ despite an urgent need for ‘emotional support to help them manage the impact of receiving the diagnosis’ in 60% of people with dementia.^11^

Existing postdiagnostic emotional support in NHS services can include genetic counselling, referrals to Improving Access to Psychological Therapies (IAPT) or the use of psychotropic medication. However, all have significant limitations. Genetic counselling is largely limited to those at risk of highly heritable dementias^12^ and people with dementia make up 0.2% of referrals to IAPT,^11^ with people with more significant impairment less likely to be treated.^13^ For psychotropic medication, there is dubious evidence for efficacy in people with dementia despite widespread use.^14^ Cognitive Stimulation Therapy is routinely offered to people with dementia,^15^ but this is a practical intervention aimed at improving cognition rather than well-being. Taken together, this suggests a gap in care provision.

Outside of clinical practice, postdiagnostic interventions for people living with dementia are often delivered by third-sector services and focus on practical interventions around decision making, future care and facilitating peer support.^16^ Research interventions for people with dementia suggest that there is growing evidence that psychosocial therapies and interventions with people with dementia are of benefit,^17^ but many fail to be scaled up for clinical practice.^18^

There are currently no standardised postdiagnostic interventions used nationally within NHS services that help a person with dementia and their carer process and adapt to a newly given diagnosis. The lack of previous interventions or focus on emotional support for adjusting to a dementia diagnosis and a lack of implementation for psychosocial therapies in dementia represents a significant barrier to the development of a new intervention. Therefore, initial development work is needed for a novel postdiagnostic intervention that can result in a meaningful impact on people living with dementia and their families. While the use of this intervention will entail cost initially, its preventative rather than reactive nature will reduce unnecessary prescribing for comorbid depression and anxiety, increase quality of life for people newly diagnosed with dementia and their supportive others, and it will reduce the burden on later NHS service use. Further, it will enable clinicians to circumvent practical barriers in IAPT services.

To develop this postdiagnostic intervention, frameworks focused on cultivating positive emotion and growth such as positive psychology approaches will be used. Positive psychology frameworks refer to the scientific study of strengths and capabilities,^19^ with evidence to suggest that diverse positive psychology interventions are associated with increases in positive affect across clinical populations^20 21^ and in people processing life- changing diagnoses.^22^ Positive psychology and related interventions are therefore ideally suited for cultivating positive emotions and personal growth in people with dementia, who actively use strengths such as hope and resilience.^23^,^26^

Systematic reviews and primary research will be conducted to develop the programme theory and understand key implementation issues. This will provide the necessary groundwork for a later programme of research, where the intervention will be refined and evaluated across NHS services. If successful, this work will generate meaningful change for people living with dementia, their families and NHS professionals.

### Aims and objectives

To develop the evidence base for a novel, postdiagnostic intervention to enhance well-being and emotional or psychological adjustment in people adjusting to a newly given dementia diagnosis and their significant others in the NHS and the local community. To accomplish this aim, the objectives are to:

1. Synthesise evidence for best practice positive psychology interventions and psychosocial interventions for people with dementia using systematic reviews.
2. Understand the implementation context including:
  - Current experience of postdiagnostic care pathways in NHS settings.
  - Wants and needs for a postdiagnostic intervention.
  - The barriers and facilitators to implementing a postdiagnostic intervention in UK NHS settings.
3. Establish an advisory group of patient and public involvement (PPI) representatives to inform and co-develop the intervention.

## Methods and analyses

**Figure 1:**
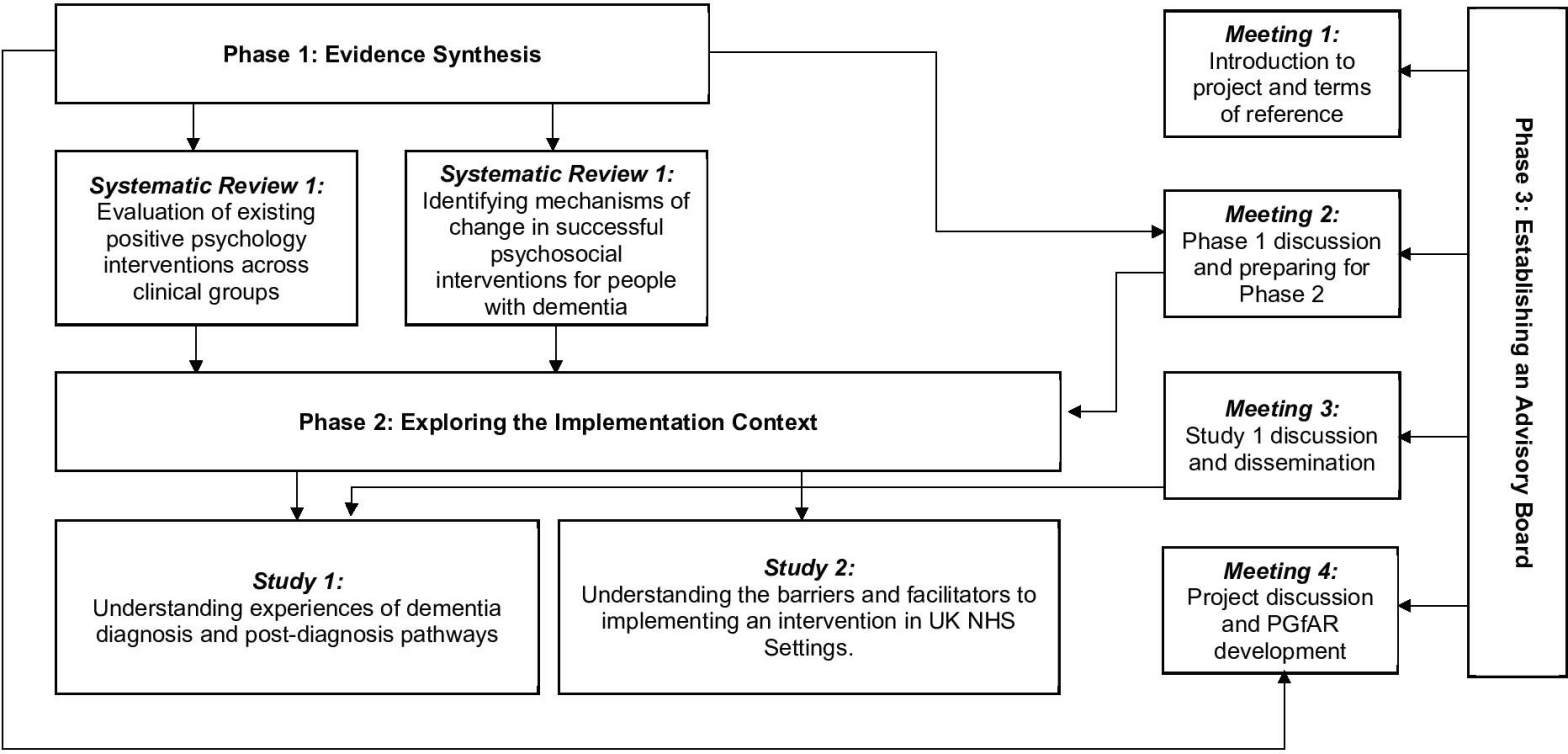
Overview of development work.

All development work will be underpinned by the recently updated Medical Research Council (MRC) guidelines for developing complex interventions^27^ and implementation theory.^28 29^ A key recommendation differentiating the 2021 guidance from the 2006 iteration is the consideration of implementation from the outset. Thus, development work will entail a consideration of the following core elements from the updated MRC framework: context, engagement of stakeholders, identification of key uncertainties and the development of a programme theory. A three-phased approach will be used to synthesise evidence and begin development work (figure 1).

### Phase 1: evidence synthesis (September 2023–September 2024)

Two systematic reviews will be undertaken by the project team. Both are PROSPERO registered^30 31^ and will conform to Preferred Reporting Items for Systematic Reviews and Meta-Analyses reporting guidelines.^32^ Systematic Review 1 (CRD42023452369) will be used to identify and appraise existing interventions where the primary outcome is a psychological or emotional adjustment to a diagnosis of a mental or physical health condition. Systematic Review 1 will also expand on previous work by including a specific focus on suitability and adaptability for people with dementia. Suitability for people with dementia will be determined by assessing whether the intervention in question has efficacy in related populations, for example, chronic illness, traumatic brain injuries and older adults.^33^ Evidence regarding the intervention’s acceptability, feasibility and cost will be synthesised and evaluated for potential use in a dementia population.

Systematic Review 2 (CRD42023463157) will be conducted concurrently to ensure the 9-month timeline for Phase 1 is feasible. Systematic Review 2 will identify existing psychosocial interventions with a robust evidence base for improving the well-being of people with dementia. As there are multiple systematic reviews and meta-analyses of psychosocial interventions for people with dementia including a systematic review of reviews,^34^ this systematic review will focus on identifying mechanisms of change in existing interventions.^35^ As such, Systematic Review 2 will identify shared mechanisms across psychosocial interventions for people with dementia that lead to an improvement in subjective well-being. The results of this review will be narratively synthesised in keeping with related systematic reviews.^36^

### Phase 2 exploring the implementation context (June 202 –September 2025)

#### *Study 1:* Understanding experiences of dementia diagnosis and postdiagnosis care pathways.

*Design:* A national open-ended survey, and in-person qualitative interviews, designed to elicit views on the lived experience of receiving a dementia diagnosis and postdiagnostic care pathways will be conducted. Data will be collected at one time point with no further requirements for participants. The use of both a national survey and local interviews is warranted as the national survey allows for a breadth of input from across the UK, while local in-person interviews allow for a more in-depth examination.

*Recruitment:* Inclusion criteria are (1) people with dementia who are within 1 year of having received a diagnosis and who reside in the community, (2) carers who self-identify as primary carers or supporters for a person living with dementia. People living with dementia in residential or nursing homes will be excluded due to the focus on the initial postdiagnostic and community-based intervention. Professional carers who are employed to provide care will be excluded. Recruitment will take place nationally using the Join Dementia Research (JDR) network and locally at an outer London NHS trust. Recruitment in JDR will prioritise rural and semirural areas, which are underserved by dementia research.^37^ Nationally, a sample size of 50 is required to identify wide-ranging experience, as survey responses tend to result in less data per question.

For local in-person interviews at the NHS trust, a sample size of 30 (15 people with dementia and 15 family carers) based on the ability to identify rare themes (10% prevalence) is anticipated.^38^ To ensure that data represent diverse experiences, local recruitment at the NHS trust will also concentrate on people from ethnic minorities, who can be under-represented in community support following a dementia diagnosis.^39^ Thus, high-concentration sampling^40^ will be used. To ensure recruitment strategies include people who are less likely to present to healthcare services, a combination of healthcare service and community recruitment will be used.

*Online survey procedure:* Participants will be advised that they can complete the survey as a person with dementia, as a carer or as a person with dementia and a carer taking part as a dyad. A welcome message on Qualtrics will first be displayed to participants to give them an overview of the survey. The welcome message will remind participants of the topic of the study, that this may be a sensitive area, and that they are free to skip any questions they do not wish to answer. Screening questions will then be used to confirm eligibility prior to participation. Participants will be asked to confirm that they are a person living with dementia, an informal carer defined as someone who provides unpaid support or care to a person living with dementia (excluding government benefits), or a person with dementia and a carer completing the survey together. They will also be asked to confirm that the person with the diagnosis received this diagnosis within the last 12 months and that the diagnosis of dementia was given in the UK. Participants will then be provided with a standardised information sheet and will have an opportunity to discuss the study with a research assistant on the telephone or during a Microsoft Teams meeting prior to their participation. They will then be asked to sign and download a digital consent form. The online survey will consist of demographic questions consisting of the diagnosis that was received (eg, Alzheimer’s disease, vascular dementia) when this diagnosis was received, age, gender, ethnicity and region of the UK where the diagnosis of dementia was received. Following this, participants will be asked to answer 13 questions over three sections: the diagnosis, coming to terms with the diagnosis, and what support was needed. Questions are both closed and open-ended, and designed to elicit views and experiences of receiving a diagnosis and the period following this. This will include emotional impact and expectations and experiences of instrumental and psychosocial support.

*Local interview procedure:* Qualitative interviews will also take place locally in-person at an outer London-based NHS trust or remotely via the telephone or Teams. Following consent, the interview will take place, or a date and time for a subsequent visit will be arranged. Participants may elect to take part in an interview in-person, remotely over video conferencing software or over the telephone. Participants may also elect to complete the interview jointly as a dyad of a person with dementia and carer.

The researcher will read a welcome message, reminding the participant what participation will involve and confirming their assent to proceed. They will also remind participants of the sensitive nature of the topic and reiterate that the participant is free to stop at any time or decline to answer a question. At this time, research staff will confirm eligibility using the screening criteria. They will also confirm the participant has not already completed the online version of this survey, to protect against cross recruitment. Following this, demographic information will be collected by the researcher. The researcher will then ask nine open-ended questions using the same three categories as the online survey.

Discussion of postdiagnostic support may result in distress, feelings of guilt, reports of negligent care and complaints. As such, all participants will be informed of the limits to confidentiality in this setting including disclosure of harm, and professional misconduct. If a disclosure is made, the Prinicpal Investigator (PI) will refer this to the relevant regulatory body. Locally, materials will be translated to maximise representation. Interview guides will be developed iteratively using findings from Phase 1 and with input from the Advisory Board. As part of the debrief information provided to all participants, they will have the opportunity to supply contact information should they wish to be kept updated as to the results of this study. This will be managed in accordance with the General Data Protection Regulations (GDPR).

*Analysis:* National survey data will be analysed using Structured Tabular Thematic Analysis ST-TA,^41^ which was developed in response to COVID-19. ST-TA is a hybrid thematic analysis for brief texts. Local interviews at the outer London NHS trust will be analysed using traditional Thematic Analysis.^42^ Analysis will focus on identifying shared experiences, both positive and negative of postdiagnostic care pathways in the UK. Further, the analysis will also seek to identify what people with dementia and carers value from a postdiagnostic intervention. One researcher will code all transcripts, and a second researcher will independently code a representative sample to check for consistency. Following this, both researchers will meet to explore themes arising from codes. When a final list of themes is created, the researchers will review and define each theme.

#### *Study 2:* Understanding the barriers and facilitators to implement a postdiagnostic intervention in UK NHS Settings.

*Design:* A qualitative study to understand the implementation context for the proposed intervention. A semistructured interview guide will be used for a series of focus groups or individual interviews. Participants will be asked their preference for either a focus group or an interview. The implementation context is UK NHS memory clinics, as they are often the final step in the diagnosis pathway.^43^

*Recruitment:* Memory Clinics at one outer London NHS trust, and local community organisations. Participants will include three groups of stakeholders.^44^ These are Group 1: policy professionals (public, private and third sector workers), decision makers, management staff in NHS services and community leaders; Group 2: healthcare professionals working in UK memory clinics, and research and development staff; Group 3: people living with dementia and carers who have presented to memory clinic services for the first time within the past year. The inclusion criterion is the current membership of one of the aforementioned groups. Between 5 and 10 stakeholders from each group will take part in one semistructured interview with a research assistant (n=30), with a minimum of 5 stakeholders in each subsample required. Recruitment from each group will be flexible, in recognition that more participants from one particular group may be needed to fully explore implementation issues. This will be monitored to identify data saturation. All participants will be asked to complete a consent form.

*Group 1 and 2 procedure:* Research staff will present the study during multidisciplinary team meetings at memory clinics to identify Group 1 and Group 2 participants. Presentations at non-NHS organisations, for example, local charities and organisations, will help identify community leaders. Research staff will be responsible for screening all potential participants against the eligibility criteria. To supplement recruitment for Group 1, publicly held email addresses for policy professionals, decision makers and community leaders will be used to identify participants. These may consist of public-, private- and third sector workers. For example, people who have participated in the development of National Institute for Health and Care Excellent (NICE) guidelines for people with dementia will be contacted. We will also use social media to advertise the research.

*Group 3 procedure:* Self-report of a dementia diagnosis is sufficient for inclusion in the study, and there is no requirement to independently verify the diagnosis using healthcare records. Clinicians may also refer participants directly to research staff, who will provide more information about the study. Participants who have not completed a consent to contact form will be asked for permission to pass on their contact details to the research team. This will be recorded in their medical notes. Research staff will also contact local non-NHS organisations, charities and community groups to advertise the research. Research staff details will be left with non-NHS organisations so that those interested can contact the team.

*All groups procedure*: Participants will be provided with a standardised information sheet. Three interview guides were adapted from previous research in which questions were developed using implementation theory^29^ and were successfully used to guide the implementation of a psychosocial intervention in diverse healthcare systems.^44 45^ Examples of questions posed include: ‘what are the known barriers people with dementia encounter when accessing postdiagnostic support?’ and ‘how are decisions about referrals for postdiagnostic support made?’ Questions were developed for each of the three groups, to focus on each group’s knowledge or experience of the implementation context. The Group 1 interview guide consists of three sections: Role of applying programmes to policy, reflection on successfully implemented interventions, and recommendations and buy-in of novel intervention. The Group 2 interview guide consists of two sections: disclosing a diagnosis and new support for people with dementia, Group 2 participants will also be asked for their interest in being trained in the novel intervention, should a later Programme Grant for Applied Research be selected for funding. If they express an interest, they will be asked for permission to store their contact details in accordance with GDPR and NHS policies. The Group 3 interview guide will consist of two sections: experiences with current support and thinking about new support.

*Analysis:* As with Study 1, all interviews will be audio-recorded, transcribed verbatim and analysed using Thematic Analysis.^42^ The analysis will focus on identifying themes associated with barriers to and facilitators of implementing a postdiagnostic intervention in UK Memory Clinics from the perspectives of the three groups of stakeholders.

### Phase 3: establishing an advisory group (September 2023–November 2025)

Recruitment: Working groups of people living with dementia such as the Dementia Engagement and Empowerment Project (DEEP) Network will be contacted to source experts by experience, who will be defined as people currently living with dementia and people who identify as a family caregiver to a person living with dementia.

Meetings: The Advisory Board will meet on four occasions over 24 months. In Meeting 1, they will be provided with an introduction to the project, including a discussion of the role of the group, and terms of reference. In Meeting 2, key findings from both systematic reviews will be presented to the group, which will coproduce recommendations for essential components of the postdiagnostic intervention. The board will also be consulted on study materials to be used in Studies 1 and 2. As part of Meeting 3, the board will discuss the results of Study 1 to contextualise findings with the lived experience of members and develop further key recommendations for the postdiagnostic intervention. This will likely include core components, modality, length and accessibility of the intervention. The board will develop communication strategies for disseminating the results of Phases 1 and 2 to the general public. Finally, in Meeting 4, the group will reflect on all development work undertaken, with a view to reaching a consensus on core components of the intervention to be further developed as part of a further research programme.

## Ethics and dissemination

### Health Research Authority application

The project was reviewed by the East of England—Essex Research Ethics Committee (REC) and was granted Health Research Authority approval on 15 July 2024 (24/EE/0126). People with dementia will take part in Studies 1 and 2. As inclusion criteria for both studies are that the person must have presented to memory clinics for the first time within the past year, it is likely that most people with dementia will have the capacity to consent to research. However, capacity assessments in line with British Psychological Society (2020) guidelines will be undertaken for all people with dementia who express an interest in participating. Capacity to consent will be treated as a process and reaffirmed at every participant contact. Participants and the Advisory Group will discuss their experience of diagnosis and postdiagnostic support. This may be compounded by COVID, which disproportionally affected older adults and people from minority ethnic groups. Discussions may result in distress or disclosures of negligent care. All discussions will be treated empathetically and sensitively. Participants will be made aware of limits to confidentiality and, if disclosures are made, the PI will refer these to the relevant regulatory body. To mitigate against ethics concerns such as these, we will work closely with our Advisory Group.

### Patient and public involvement

People living with dementia were consulted during the development of this work to ensure the proposed work best represented what they needed from research. Feedback was sought from members of the DEEP Network, who provided the team with examples of their own experiences of postdiagnostic support. Further, PPI will continue to be a fundamental component of development work, and our Advisory Board comprising of people living with dementia and carers will lead all PPI activities. The PPI Advisory Board will meet at regular intervals over the study period and advise on study design and materials, discuss and contextualise results from each phase, work with the research team to develop appropriate dissemination opportunities and communication strategies and answer key questions regarding the design of the postdiagnostic intervention.

### Dissemination

Phase 1 outputs will include two systematic reviews. Both are PROSPERO registered and will be submitted for publication in high-impact international journals. Phase 2 consists of two qualitative research studies, which will be submitted to relevant research journals. The study team and Advisory Board will work closely together to identify opportunities to disseminate findings outside of academic outputs, including liaising with networks such as the DEEP.
