# Supplementary material for: No one left to cope alone (NOLA): protocol for the development of a postdiagnostic intervention to support people newly diagnosed with dementia
Source: BMJ Open. 2024 Oct 11;14(10):e091241. doi: 10.1136/bmjopen-2024-091241 (PMC11474887; doi:10.1136/bmjopen-2024-091241)
Supplement: Supplementary file 1 [file bmjopen-2024-091241.reviewer_comments.pdf]

PEER REVIEW HISTORY

BMJ Open publishes all reviews undertaken for accepted manuscripts. Reviewers are asked to complete a checklist review form (**http://bmjopen.bmj.com/site/about/resources/checklist.pdf**) and are provided with free text boxes to elaborate on their assessment. These free text comments are reproduced below.

ARTICLE DETAILS

**TITLE (PROVISIONAL)**	No-one left to cope alone (NOLA): protocol for the development of a post-diagnostic intervention to support people newly diagnosed with dementia.
**AUTHORS**	Stoner, Charlotte; Durgante, Helen; Birt, Linda; Spector, Aimee

This article was not externally reviewed at BMJ Open. Protocols that have been independently assessed prior to submission to BMJ Open are usually fast-tracked to publication on the grounds that further substantial changes will not be possible. This independent assessment will usually be external, independent review for both a substantial grant award from a non-commercial or government funder and ethics approval.
